# Fine Particulate Air Pollution and Hospital Admissions for Chronic Obstructive Pulmonary Disease: A Case-Crossover Study in Taipei

**DOI:** 10.3390/ijerph10116015

**Published:** 2013-11-11

**Authors:** Shang-Shyue Tsai, Chih-Ching Chang, Chun-Yuh Yang

**Affiliations:** 1 Department of Healthcare Administration, I-Shou University, Kaohsiung 824, Taiwan; E-Mail: sionghak@isu.edu.tw; 2 Department of Environmental and Occupational Health, National Cheng Kung University, Tainan 701, Taiwan; E-Mail: chang3@mail.ncku.edu.tw; 3 Department of Public Health, College of Health Sciences, Kaohsiung Medical University, Kaohsiung 807, Taiwan; 4 Division of Environmental Health and Occupational Medicine, National Health Research Institute, Miaoli 350, Taiwan

**Keywords:** fine particulate, air pollution, COPD, case-crossover, hospital admissions

## Abstract

We undertook this study to investigate whether there is an association between atmospheric fine particles (PM_2.5_) levels and inpatient admissions for chronic obstructive pulmonary disease (COPD) in Taipei, Taiwan. Data on inpatient admissions for COPD and ambient on air pollution levels in Taipei were obtained for years 2006 to 2010. We estimated the relative risk of inpatient admissions for COPD using a case-crossover design with the following control variables: weather measures, day of the week, seasonality, and long-term time trends. For the single-pollutant model (not controlling for other atmospheric pollutants), COPD admissions were significantly and positively associated with higher PM_2.5_ levels during both warm days (>23 °C) and cool days (<23 °C), with an interquartile range increase of 12% (95% CI = 8–16%) and 3% (95% CI = 0–7%) in COPD admissions, respectively. In the two-pollutant models, PM_2.5_ remained significant even controlling for SO_2_ or O_3_ on both warm and cool days. Taken as a whole, our study demonstrates that higher levels of PM_2.5_ may increase the risk of inpatient admissions for COPD.

## 1. Introduction

Over the past decade, many epidemiologic studies demonstrated positive associations between ambient levels of airborne particulate matter (PM) (generally measured as PM with an aerodynamic diameter <10 um (PM_10_)) increased mortality [1,2,3,4,5,6,7] and hospital admissions or emergency room (ER) visits for cardiovascular and respiratory morbidity [8,9,10,11,12]. The evidence on adverse effects of PM air pollution on public health has led to more stringent standards for levels of PM in outdoor air in the USA and in other countries [13,14]. 

While previous studies have primarily used PM_10_ as an exposure indicator, fine particles (defined as PM with an aerodynamic diameter <2.5 um; PM_2.5_) have become a greater health and regulatory concern due to epidemiologic studies suggesting that PM_2.5_ might exert greater toxicity than larger particles [15,16,17,18]. It is now generally accepted that PM_2.5_ are more harmful for health than larger particles (PM_10_) because PM_2.5_ can be inhaled more deeply into the lungs and offer a greater surface area and hence potentially larger concentrations of adsorbed or condensed toxic air pollutants per unit mass [19,20]. It is for this reason exactly that the World Health Organization (WHO) recommends using PM_2.5_ instead of PM_10_ concentrations as indicators of air quality [6].

Relatively few epidemiologic studies have been undertaken which specifically address the health effects of PM_2.5_ due to lack of PM_2.5_ monitoring data [21]. Considerable attention has been focused on all respiratory admissions [17,21,22,23,24,25], possibly combining outcomes with different sensitivities to air pollution and different lags between exposure and hospitalization [26]. Several studies investigated the relationship between PM_2.5_ and hospital admissions (or ER visits) for chronic obstructive pulmonary disease (COPD) [13,23,27,28,29,30]. These studies were conducted principally in North America and European cities, so the findings may not be generalized to Taiwan, where the air pollutant mixtures may potentially be quite different. 

There is a substantial number of epidemiological studies in Taiwan reporting associations between mortality/morbidity with ambient PM_10_ levels [31,32,33,34,35,36], but only a limited number of studies evaluating associations with PM_2.5_ levels, due primarily to the lack of monitoring data [37,38,39,40,41]. 

Our primary objective was to examine the short-term impact of PM_2.5_ levels on daily inpatient admissions for COPD among residents of Taipei, the largest city in Taiwan, using over 5 years of observations from 2006–2010, and a case-crossover design.

## 2. Materials and Methods

### 2.1. Taipei City

This study examined daily variations in hospital admissions for COPD in relation to PM_2.5_ levels in Taipei for the 5-year period from 2006 through 2010. Taipei is the largest metropolitan city in Taiwan with a population of approximately 2.64 million located in northern Taiwan. The major air pollution source is automobile exhaust emission. Taipei has a subtropical climate, with an annual average temperature of 23 °C. (the months with a mean temperature below 23 °C are from November to April and May to October are the months with a mean temperature above 23 °C).

### 2.2. Hospital Admission Data

The National Health Insurance (NHI) Program, which provides compulsory universal health insurance, was implemented in Taiwan on 1 March 1995. Under the NHI, 98% of the island’s population receives all forms of health care services including out-patient services, in-patient care, Chinese medicine, dental care, childbirth services, physical therapy, preventive health care, home care, and rehabilitation for chronic mental illness. Most medical providers (93%) were under contract with the Bureau of NHI (BNHI), and those not under contract provide fewer health services. More than 96% of the population with insurance coverage through the NHI utilized health services at least one time at contracted medical institutions. 

Data in electronic format on daily clinic visits or hospital admissions can be obtained for each contracted medical institution. In order to be reimbursed, all medical institutions must submit their claims for billable medical services on a standardized electronic form, which includes data elements such as the date of admission and discharge, patient identification number, gender, birthday, and the ICD-9-CM diagnostic code for each admission. Therefore, the information from the NHI database appears to be sufficiently complete, reliable, and accurate for use in epidemiological studies. Daily counts of hospital admissions with a primary diagnosis of COPD (International Classification of Diseases, 9th revision [ICD-9] codes 490, 492, 494, and 496) were extracted from the medical insurance files for the period 2006–2010.

### 2.3. PM_2.5_ and Meteorological Data

Six air quality monitoring stations were established in Taipei city by the Taiwanese Environmental Protection Administration (EPA), a central governmental agency in 1994. The monitoring stations were fully automated and routinely monitored five “criteria” pollutants including sulfur dioxide (SO_2_) (by ultraviolet fluorescence); particulate matter (PM_10_) (by beta-ray absorption); nitrogen dioxide (NO_2_) (by ultraviolet fluorescence), carbon monoxide (CO) (by nondispersive infrared photometry), and ozone (O_3_) (by ultraviolet photometry) levels. However, PM_2.5_ was not regularly monitored. PM_2.5_ concentrations in Taiwan were measured continuously since 2006. PM_2.5_ was measured using tapered element oscillating microbalance method samplers. The availability of the monitoring network for PM_2.5_ provided an opportunity to investigate the impact of PM_2.5_ on hospital adsmissions for COPD. For each day, hourly air pollution data were obtained for all of the monitoring stations. After calculating the hourly mean of each pollutant from the six stations, the 24 h average levels of these pollutants were computed. Daily information on mean temperature and mean humidity was provided by the Taipei Observatory of the Central Weather Bureau.

### 2.4. Statistics

Data were analyzed using the case-crossover technique [42,43,44]. This design is an alternative to Poisson time series regression models for studying the short-term effects attributed to air pollutants [45]. In general, the case-crossover design and the time-series approach yielded almost identical results [46,47,48]. 

For our case-crossover analysis, we adopted a time-stratified approach [45]. We stratified time into separate months in order to select referent days falling on the same day of the week within the same month as the index day. Air pollution levels during the case period were compared with exposures occurring on all referent days. This time-stratified referent selection scheme minimizes bias due to non-stationarity of air pollution time-series data [49,50,51]. The results of previous studies indicated that increased number of hospital admissions were associated with higher air pollutant levels on the same day or the previous two days [52]. Longer lag times have rarely been described. Thus the cumulative lag period up to 2 previous days (*i.e.*; the average air pollutant levels of the same and previous 2 days) was used. Because pollutants vary considerably by season, especially O_3_ and particles, seasonal interactions between PM_2.5_ and hospital admissions have often been reported. However, previous studies were conducted mostly in countries where the climates are substantially different from that in Taipei [31,35], which has a subtropical climate with no apparent 4-season cycle. Hence in this study the potential interactions of seasonality on the effects of PM_2.5_ was not considered; but temperature was used instead. The adverse health effects of each air pollutant were examined for the “warm” days (days with a mean temperature above 23 °C) and “cool” days (days with a mean temperature below 23 °C) separately. 

The associations between hospital admissions for COPD and levels of PM_2.5_ were estimated using the odds ratio (OR) and their 95% confidence intervals (CI) which were produced using conditional logistic regression with weights equal to the number of hospital admissions on that day. All statistical analyses were performed using the SAS package (version 9.2; SAS Institute, Inc.; Cary, NC, USA). Both single-pollutant models and multi-pollutant models were fitted with a different combination of pollutants (up to two pollutants per model) to assess the stability of the effect of PM_2.5_. Exposure levels to air pollutants were entered into the models as continuous variables. Meteorologic variables such as daily average temperature and humidity on the same day, which might play a confounding role, were included in the model. In all analyses, we modeled the mean temperature at lag 0 as a quadratic function, mean humidity at lag 0, and pollutants as a linear function of the 3-day moving average of current and previous 2 days concentrations (lag 0–2). Inclusion of barometric pressure did not markedly change the effect estimates and therefore was not considered in the final model. OR were calculated for the interquartile difference (IQR, between the 25th and the 75th percentile) for PM_2.5_, as observed during the study period.

## 3. Results and Discussion

During the 5 years of the study, there were a total of 22,424 hospital admissions for COPD for the 47 hospitals in Taipei city. The descriptive statistics for admissions and corresponding environmental data are shown in Table 1. There was an average of 12.28 daily hospital admissions for COPD in the city over the study period. 

Pearson’s correlation coefficients among the air pollutants are presented in Table 2. Significant cross-correlations among pollutants were observed, especially between PM_10_ and PM_2.5_ (*r* = 0.78), PM_2.5_ and SO_2_ (*r* = 0.61), PM_2.5_ and NO_2_ (*r* = 0.54), PM_2.5_ and CO (*r* = 0.54), SO_2_ and NO_2_ (*r* = 0.52), SO_2_ and CO (*r* = 0.50), and between NO_2_ and CO (*r* = 0.89). 

Table 3 shows the effect estimates of PM_2.5_ on hospital admissions for COPD in single-pollutant models and two-pollutant models. For the single pollutant model (without adjustment for other pollutants), increased admissions for COPD were significantly associated with PM_2.5_ both on warm (>23 °C) and cool days (<23 °C), with an IQR increase associated with a 12% (95% CI = 8–16%) and 3% (95% CI = 0–7%) increase in COPD admissions, respectively. In two-pollutant models, PM_2.5_ remained significant after the inclusion of SO_2_ or O_3_ both on warm and cool days.

This study is one of the few that investigated the association between exposure to PM_2.5_ and hospital admissions for COPD and is the first in Asia. Data demonstrated that the levels of PM_2.5_ were positively associated with increases in the daily number of hospitalizations for COPD after inclusion of SO_2_ or O_3_ both on warm and cool days. The observed effects of PM_2.5_ were not maintained in the presence of NO_2_ or CO. It is possible that the effect of PM_2.5_ might have been masked by those of NO_2_ and CO. 

Studies on the effect of PM_2.5_ on COPD admissions are rare. Belleudi *et al.* conducted a study in Rome, and found no evidence of an association between COPD admissions and exposure to PM_2.5_ [28]. A study in Helsinki by Halonen *et al.* [23] reported a 5.3% increase in the risk of COPD admission per 10 ug/m^3^ increase in the level of PM_2.5_ which is similar to their study results on COPD ER visits (4.6% for 10 ug/m^3^ PM_2.5_) [27]. Dominici *et al.* reported a 1.61% (95% CI = 0.56–2.66%) increase in hospitalization for COPD per 10 ug/m^3^ increase in PM_2.5_ level [13]. In this study, we found a 6.87% (which corresponds to 12% increase per IQR increment) and 1.72% (which corresponds to 3% increase per IQR increment) increase in hospitalization for COPD per 10 ug/m^3^ increment in the 3 day moving average (lag 2) concentrations of PM_2.5_ for warm days and cool days, respectively.

In our study, effects were observed on both warm and cool days, but they were larger on warm days (effect modification). We were able to confirm that PM effects vary by season [17,22]. The observed seasonal variation in effect estimates could be explained by variation in exposure patterns. People in Taipei are more likely to go outdoors and open the windows in the warm season than in cool season (higher exposure); thus, monitored PM_2.5_ concentrations may be closer to personal exposure to PM_2.5_ in the warm season than in the cool season (better exposure assessment). The fact may attenuate the PM_2.5_ effect in the cool season. On the other hand, seasonal differences in air pollution mixture may also affect the effect estimates. Furthermore, compared with other studies in developed countries, our study found larger effect estimates per unit increase of PM_2.5._ One potential explanation for this discrepancy is that most published studies only demonstrated straightforward pooled effect estimates (lack of effect estimates stratified by season). This may conceal inherent differences between different climates and air pollution mixtures. Variations in seasonal and regional effect estimates may in part result from differences in the chemical composition of PM_2.5_ [22]. Nevertheless, the seasonal pattern of air pollution health effects need to be further investigated.

The most common and consistent associations between air pollutants and hospital admissions for respiratory disease were found with PM [53]. A significant association was found between PM_2.5_ exposure and COPD admissions in this study. This finding is in agreement with previous studies [13,23,27]. Some pathophysiological hypotheses have been proposed to explain the association between short-term effects of PM_2.5_ and COPD admissions. Researchers have suggested that PM_2.5_ levels represent the effective toxic fraction of PM, because PM increase and sustain oxidative stress both on the entire respiratory tract and on the systemic level, where oxidative stress induces inflammation [54,55]. In the lungs, PM may cause inflammation and thereby aggravate an underlying lung disease, thereby reducing the efficacy of lung-defense mechanisms [13]. Animal studies have shown an increased vulnerability to PM in animal with cardiopulmonary disease [56], and exacerbations of ongoing pneumococcal infection after exposure to concentrated ambient PM_2.5_ [57].

Numerous studies have documented an association between air pollution and increased hospital admissions in cities worldwide. Major PM_2.5_ components vary geographically seasonally, but typically include ammonium sulfate and nitrate, elemental carbon, carbonaceous species, carbonates, metals, and water [24,58]. Despite the volume of research on the subject, the relative toxicity of different components of PM_2.5_ remains unanswered but toxicity is likely to vary between components [58].

Maclure proposed the case-crossover study design as a way to study the influence of transient, intermittent exposures on the subsequent risk of uncommon acute-onset events shortly after exposure [42]. This design offers the ability to control many confounders by design rather than by statistical modelling. This design is an adaptation of the case-control study in which each case serves as his or her own referent. Therefore time-invariant subject-specific variables such as gender, age, underlying chronic disease, or other individual-level characteristics do not act as confounders. In addition, time-stratified approach [43] was found to be effective in controlling for seasonality, time trends, and chronic and slowly varying potential confounders [49,50,51]. In general, the case-crossover design and the general additive model (GAM) approach, which has been the analytic method of choice for studying short-term adverse effects of air pollution since 1990 [59], produced almost identical results [46,47,48]. 

For a factor to confound the relationship between PM_2.5_ levels and admissions for COPD it needs to be correlated with both variables. It is unlikely that smoking and other indoor pollutants confound the present association since day to day variations in indoor emissions, including smoking may not be correlated with PM_2.5_ air pollution. 

Exposure measurement error is a common concern in environmental epidemiology. PM_2.5_ levels were assigned from fixed, outdoor monitoring stations to individuals to estimate exposure (assuming that exposure was homogeneous over all the studied area). Exposure measurement errors resulting from differences between the population‑average exposure and ambient PM_2.5_ levels are not avoidable. This kind of measurement error will result in nondifferential misclassification. However, this exposure misclassification is likely to cause a bias toward the null and lead to underestimates of pollutant effects [52,60]. 

Our study population is homogenous in terms of race compared with populations in other cities. This study was conducted in a subtropical city. These facts may restrict somewhat the generalizability of these findings to other locations with different meteorological and racial characteristics. Further, behavior such as air conditioning usage or time spent outdoors may affect personal exposures. This might affect the magnitude of the observed associations compared with other geographical locations.

**Table 1:** Distribution of daily COPD admissions, weather, and air pollution variables in Taipei, Taiwan, 2006–2010.

Variable ^a^	Min	Percentile			Max	Mean	Days
		25%	50%	75%			
PM_10_ (ug/m^3^)	14.26	34.89	46.83	62.37	888.02	51.71	1,826
PM_2.5_ (ug/m^3^)	8.35	19.46	27.06	36.92	140.54	29.99	1,826
SO_2_ (ppb)	1.00	2.73	3.65	4.91	11.14	3.94	1,826
NO_2_ (ppb)	3.22	19.97	23.86	28.81	55.59	24.67	1,826
CO (ppm)	0.13	0.50	0.63	0.80	1.76	0.68	1,826
O_3_ (ppb)	4.00	17.95	23.95	30.23	70.89	24.65	1,826
Temperature(°C)	9.35	19.50	24.11	28.42	33.18	23.69	1,826
Humidity (%)	31.37	66.54	73.11	79.57	94.19	72.82	1,826
COPD admissions	0	9	12	15	30	12.28	1,826

Abbreviation: Min, minimum value; Max, maximum value; ^a^ 24 h average.

**Table 2:** Correlation coefficients among air pollutants.

Variable	PM_10_	PM_2.5_	SO_2_	NO_2_	CO	O_3_
PM_10_	1.0	0.78	0.43	0.35	0.35	0.26
PM_2.5_	–	1.0	0.61	0.54	0.54	0.31
SO_2_	–	–	1.0	0.52	0.50	0.06
NO_2_	–	–	–	1.0	0.89	−0.07
CO	–	–	–	–	1.0	−0.23
O_3_	–	–	–	–	–	1.0

**Table 3:** Association between PM_2.5_ and admissions for COPD in Taipei, Taiwan, 2006–2010.

Temperature		PM_2.5_ OR (95% CI) ^a^
≥23 °C (1,021 days)	Without adjustment ^b^	1.12 (1.08–1.16)
	Adjusted for SO_2_	1.14 (1.09–1.19)
	Adjusted for NO_2_	1.02 (0.97–1.07)
	Adjusted for CO	1.01 (0.96–1.05)
	Adjusted for O_3_	1.12 (1.08–1.17)
<23 °C (805 days)	Without adjustment ^b^	1.03 (1.00–1.07)
	Adjusted for SO_2_	1.12 (1.07–1.17)
	Adjusted for NO_2_	0.97 (0.94–1.01)
	Adjusted for CO	1.02 (0.98–1.06)
	Adjusted for O_3_	1.04 (1.01–1.08)

^a^ Calculated for an interquartile range increases of PM_2.5_ (17.46 ug/m^3^) and adjusted for temperature and humidity; ^b^ Single pollutant model.

## 4. Conclusions

In summary, this study provided evidence of associations between short-term exposure to PM_2.5_ and increased hospital admissions for COPD.
